# Evolutionary Patterns of Thylakoid Architecture in Cyanobacteria

**DOI:** 10.3389/fmicb.2019.00277

**Published:** 2019-02-22

**Authors:** Jan Mareš, Otakar Strunecký, Lenka Bučinská, Jana Wiedermannová

**Affiliations:** ^1^Center Algatech, Institute of Microbiology, Czech Academy of Sciences, Třeboň, Czechia; ^2^Department of Aquatic Microbial Ecology, Institute of Hydrobiology, Biology Centre, Czech Academy of Sciences, České Budějovice, Czechia; ^3^Faculty of Science, University of South Bohemia, České Budějovice, Czechia; ^4^Institute of Aquaculture, Faculty of Fisheries and Protection of Waters, University of South Bohemia, České Budějovice, Czechia; ^5^Laboratory of Molecular Genetics of Bacteria, Institute of Microbiology, Czech Academy of Sciences, Prague, Czechia

**Keywords:** cyanobacteria, evolution, photosynthesis, phylogenomics, thylakoid pattern, SSU rRNA gene, taxonomy

## Abstract

While photosynthetic processes have become increasingly understood in cyanobacterial model strains, differences in the spatial distribution of thylakoid membranes among various lineages have been largely unexplored. Cyanobacterial cells exhibit an intriguing diversity in thylakoid arrangements, ranging from simple parietal to radial, coiled, parallel, and special types. Although metabolic background of their variability remains unknown, it has been suggested that thylakoid patterns are stable in certain phylogenetic clades. For decades, thylakoid arrangements have been used in cyanobacterial classification as one of the crucial characters for definition of taxa. The last comprehensive study addressing their evolutionary history in cyanobacteria was published 15 years ago. Since then both DNA sequence and electron microscopy data have grown rapidly. In the current study, we map ultrastructural data of >200 strains onto the SSU rRNA gene tree, and the resulting phylogeny is compared to a phylogenomic tree. Changes in thylakoid architecture in general follow the phylogeny of housekeeping loci. Parietal arrangement is resolved as the original thylakoid organization, evolving into complex arrangement in the most derived group of heterocytous cyanobacteria. Cyanobacteria occupying intermediate phylogenetic positions (greater filamentous, coccoid, and baeocytous types) exhibit fascicular, radial, and parallel arrangements, partly tracing the reconstructed course of phylogenetic branching. Contrary to previous studies, taxonomic value of thylakoid morphology seems very limited. Only special cases such as thylakoid absence or the parallel arrangement could be used as taxonomically informative apomorphies. The phylogenetic trees provide evidence of both paraphyly and reversion from more derived architectures in the simple parietal thylakoid pattern. Repeated convergent evolution is suggested for the radial and fascicular architectures. Moreover, thylakoid arrangement is constrained by cell size, excluding the occurrence of complex architectures in cyanobacteria smaller than 2 μm in width. It may further be dependent on unknown (eco)physiological factors as suggested by recurrence of the radial type in unrelated but morphologically similar cyanobacteria, and occurrence of special features throughout the phylogeny. No straightforward phylogenetic congruences have been found between proteins involved in photosynthesis and thylakoid formation, and the thylakoid patterns. Remarkably, several postulated thylakoid biogenesis factors are partly or completely missing in cyanobacteria, challenging their proposed essential roles.

## Introduction

Emergence of oxygenic photosynthesis in cyanobacteria was one of the most important milestones in the evolution of life on Earth. It facilitated formation of oxygen-rich atmosphere (Schirrmeister et al., 2015, 2016) and boosted the evolution of eukaryotic algae and plants by endosymbiotic formation of chloroplasts (Yoon et al., 2004; Keeling, 2013). Cyanobacterial photosynthesis continues to contribute significantly to the oxygen and carbon cycles, mainly owing to vast abundance of simple picocyanobacteria in oceans (Flombaum et al., 2013).

Cyanobacteria and photosynthetic organelles derived from them utilize intracellular thylakoid membranes to harbor the photosynthetic apparatus (phycobilisomes/light harvesting complexes, photosystems) and to generate the transmembrane proton gradient necessary for ATP synthesis (Järvi et al., 2013; Mullineaux, 2014). Thylakoids are membrane vesicles capable of forming elaborate networks, which are however believed to enclose a single lumenal space (van de Meene et al., 2006; Ting et al., 2007; Liberton et al., 2011a,b; Pribil et al., 2014). A single known example of an oxygenic phototroph lacking thylakoids is the phylogenetically basal unicellular thylakoid-less cyanobacterium *Gloeobacter* (Rippka et al., 1974; Mareš et al., 2013). In this organism, the photosynthetic complexes (specifically phycobilisomes) were demonstrated to be attached directly to the inner surface of the plasma membrane (Guglielmi et al., 1981), similar to anoxygenic phototrophic bacteria.

The exact course of thylakoid biogenesis remains a matter of debate (reviewed by Rast et al., 2015). Although the lipid composition of the photosynthetic membrane is highly specific (Boudiére et al., 2014), in plants, the thylakoid membrane seems to be initially formed by invagination of the inner chloroplast envelope, which descends from cellular membrane of the cyanobacterial endosymbiont. This process is followed by vesicular transport of specific lipids into mature chloroplasts (Kowalewska et al., 2016). The biogenesis process further requires a highly concerted assembly of numerous proteins and their co-factors that can cover more than 70% of the thylakoid membrane surface (Kirchhoff et al., 2002). Several proteins such as Vipp1, CurT, YidC/Alb3, and TerC were postulated to be involved in thylakoid biogenesis (Chigri et al., 2012), although their role is not clear and may not be directly connected to the formation of the membrane itself (Zhang et al., 2014). In cyanobacteria, the thylakoid membrane biogenesis is less understood. After decades of research, focused mainly on assembly of photosynthetic complexes, it is assumed that there is a specialized micro-domain or active assembly place which is responsible for putting all the components such as pigments, cofactors, and proteins together (Nickelsen et al., 2011; Nickelsen and Zerges, 2013). Yet the exact localization in the cell remains enigmatic and only indirect evidence is available concerning the place of the step-wise assembly process (Komenda et al., 2012; Rast et al., 2015). So-called “thylakoid centers” observed close to the plasma membrane at sites of thylakoid membrane convergence were described in various cyanobacterial species (Kunkel, 1982) and characterized as rod-shaped structures protruding through cells of the model strain *Synechocystis* sp. PCC 6803 (van de Meene et al., 2006). The thylakoid centers seem to be closely associated with both the plasmatic and the thylakoid membrane systems; hence, it is tempting to speculate that they play a role in the biogenesis of the thylakoids (Rast et al., 2015).

The architecture of the thylakoid membrane system is variable among chloroplasts and cyanobacteria. In plants and some of the green algae, chloroplasts contain two distinct types of thylakoid membrane structures, grana and stroma lamellae. The grana are vertically stacked discoid membranes, interconnected into a continuous thylakoid network by the non-appressed stroma lamellae (Mustardy and Garab, 2003; Shimoni et al., 2005). Photosynthetic protein complexes are unequally distributed between the two membrane structures, which corresponds to their morphological and functional differentiation (Pribil et al., 2014). The lateral heterogeneity in protein organization, interaction between the proteins (in particular mediated by light harvesting complex II), and effect of hydrogen bonds between the headgroups of membrane glycolipids likely contribute to the observed grana stacking (Standfuss et al., 2005; Demé et al., 2014; Yokoyama et al., 2016). Recently an important role in thylakoid morphology has been proposed for CurT1. This protein is highly abundant within grana margins where it likely controls their curvature (Armbruster et al., 2013). CurT1 homologs exist also in cyanobacteria in which they likewise contribute to membrane shaping as evidenced in CurT^-^ mutant *Synechocystis* sp. PCC 6803 (Heinz et al., 2016). Nevertheless, its specific role in the thylakoid structure has not yet been experimentally tested.

Cyanobacterial thylakoids do not form grana, yet their architecture is not always simple. Since the implementation of transmission electron microscopy (TEM) in bacteriology, the variability in their spatial arrangement has been repeatedly recognized (Ris and Singh, 1961; Lang, 1968; Gantt and Conti, 1969; Cohen-Bazire, 1988; Komárek and Kaštovský, 2003; Gonzalez-Esquer et al., 2016). The molecular mechanisms responsible for this diversity of shapes remain unknown. Thylakoid ultrastructure has been proposed to provide conserved features that could be used for classification (Whitton, 1972; Hoffmann, 1988; Komárek and Čáslavská, 1991). Cyanobacterial taxonomists have widely adopted a polyphasic approach that combines genetic and phenotypic evidence to establish monophyletic taxa characterized by unique apomorphies (Castenholz and Norris, 2005; Johansen and Casamatta, 2005; Dvořák et al., 2015). In this framework, thylakoid arrangement has been utilized as one of the crucial pieces of information (Castenholz, 2001; Hoffmann et al., 2005; Komárek et al., 2014).

The last systematic survey focusing on the evolution of thylakoid arrangement in cyanobacteria was published 15 years ago (Komárek and Kaštovský, 2003). Based on phylogenies inferred from the SSU rRNA gene and TEM photographs of cyanobacterial strains, the authors pointed out that thylakoid patterns seem to be conserved in selected groups. The parietal (peripheral) arrangement of thylakoids was typically seen in phylogenetically basal and morphologically simple coccoid and filamentous cyanobacteria, nowadays placed in Synechococcales (Komárek et al., 2014). An arrangement with thylakoids protruding from the cell periphery to its center forming a radial pattern was observed in the families Phormidiaceae and Oscillatoriaceae (derived unbranched filamentous morphotypes) as well as few coccoid cyanobacteria, especially *Cyanothece sensu stricto*. A special arrangement with parallel thylakoids filling the entire cell in longitudinal section was found in the genus *Cyanobacterium*. The last type recognized by Komárek and Kaštovský (2003) exhibited irregularly coiled “wavy” thylakoids, and included heterocytous cyanobacteria (Nostocales) together with several coccoid ones (e.g., *Chroococcus*). The authors speculated about possible evolutionary routes connecting the various types of thylakoid arrangement. However, the phylogenetic trees in their study were quite simple, did not include branch support or ancestral state analyses, and the taxon sampling at that time was rather restricted.

Vast majority of DNA sequence data, including thousands of SSU rRNA gene sequences and hundreds of genome sequences of cyanobacteria, has been collected in the past decade. Simultaneously, TEM analyses became a regular part of polyphasic studies and fine structure of numerous strains has been reported. In the present study, we capitalize on such wealth of new information in order to test and further develop the hypotheses about connection between phylogeny and thylakoid pattern in cyanobacteria postulated first by Komárek and Kaštovský (2003). We complement the published structural data with our new ultrastructural images and bioinformatic analyses to construct a robust phylogenomic tree to overcome the problems caused by single-gene SSU rRNA phylogenies (Mareš, 2018) and to embed the evolution of thylakoid morphology in the current genomic context. This allowed us to propose a simplified evolutionary scheme for thylakoid architecture in cyanobacteria. Despite this we find that thylakoid arrangement alone is seldom a good taxonomic indicator and that many of the genes previously proposed to be essential for thylakoid morphogenesis are missing in some species.

## Materials and Methods

### TEM and Morphometric Analysis

The studied strains (Supplementary Table S1) were purchased from public culture collections and cultivated in liquid and agar-solidified BG11 medium (Rippka et al., 1979) at 20°C and 16:8 light:dark cycle. For ultrastructural studies, biological material of cyanobacteria was harvested by centrifugation (5 min at 3000 ×*g*) after 2–4 weeks of cultivation (the late exponential/early stationary growth phase), fixed with 2.5% glutaraldehyde in 0.1 M phosphate buffer at pH 7.2 (PB) and kept at 4°C temperature overnight. Followed by washing steps with the 4% glucose in PB, samples were post-fixed with 1% osmium tetroxide in the same buffer at room temperature for 3 h. After being washed again in the same buffer, cyanobacteria were pelleted and embedded in 1% agarose, cut into small cubes that were dehydrated through a graded series of acetone (30, 50, 70, 80, 90, 95, 100% 3×) for 15 min each, embedded in low-viscosity Spurr resin (EMS, Spurr, 1969) and polymerized at 60°C for 48 h. Ultrathin sections of 60–70 nm were cut using an ultramicrotome (UCT, Leica). Sections were collected on Formvar-coated copper grids and stained with saturated uranyl acetate in 50% ethanol for 30 min followed by Reynold’s lead citrate for 20 min (Reynolds, 1963). Prepared sections were examined in a JEOL 1010 transmission electron microscope (JEOL) operating at 80 kV equipped with a Mega View III camera (SIS).

Morphological data were assembled from thylakoid arrangements and cell dimensions reported in previously published papers and TEM photographs from both previously published and the current study (Supplementary Table S1). Morphometric variables included: (i) the smallest cell dimension, (ii) the largest cell dimension, (iii) the medium smaller cell dimension, and (iv) the medium larger cell dimension. The latter two were assessed as the mid value of the reported (or measured) range of the dimension, excluding rare extreme values. For few strains photographs of only a single or several cells were available which caused the smallest/mid-smaller and the largest/mid-larger dimensions to be the same. Types of thylakoid arrangement were divided into eight categories based on visual estimation of the TEM data set and, where possible, in accordance with the categories created by Komárek and Kaštovský (2003) to allow direct comparison: (i) thylakoids absent, (ii) parietal, (iii) radial, (iv) fascicular (wavy), (v) parallel, (vi) irregular, (vii) *Cyanothece* type, and (viii) unknown or equivocal. Several special but repeatedly observed features in thylakoid arrangement were further categorized as follows: (i) no special feature, (ii) triangular structure, (iii) parietal arrangement with fascicles protruding into the center of the cell, (iv) spherical formations, and (v) concentric arrangement with a layer of appressed thylakoids. Further explanation and examples of these categories are provided in the section “Results.” The raw morphometric data are listed in Supplementary Table S1. Variance in morphometric variables among the main categories of thylakoid arrangement was assessed using Dell Statistica v 13 (Dell Inc., 2016) employing Kruskal–Wallis test, with pair-wise comparisons between the categories evaluated by standard two-tailed *t*-tests.

### Phylogenetic Analysis of the SSU rRNA Gene

A set of 150 SSU rRNA gene sequences spanning over majority of the known major cyanobacterial lineages was assembled based on literature search for all available isolates that have been both sequenced and analyzed using TEM. Additionally, 148 complete SSU rRNA gene sequences were mined from the set of selected high-quality genome assemblies of cyanobacteria and several outgroup bacteria (the selection is described later on in the section “Phylogenetic Analysis of Housekeeping Loci”) to allow a later comparison of phylogenetic clades between the SSU rRNA and the phylogenomic tree. A single rRNA operon copy per genome was used because significant differences in phylogenetic clustering among the copies were not expected (Mareš et al., 2018). The final set of 298 sequences was aligned using MAFFT v. 7 (Katoh and Standley, 2013), and manually corrected. The resulting alignment covered nearly the entire SSU rRNA gene and spanned 1477 positions after ambiguous gap sites were removed.

The best nucleotide substitution model for phylogenetic analysis was selected using jModelTest 2 software (Darriba et al., 2012) based on the Akaike Information Criterion: general time-reversible model with invariant sites and gamma distribution of rates (GTR+I+G). The phylogenetic reconstruction applying this substitution model was conducted using Bayesian inference (BI) in MrBayes 3.2.6 (Ronquist et al., 2012). The BI calculation employed Metropolis-Coupled Markov Chain Monte Carlo (MCMCMC) analyses with seven heated chains and one cold chain in each of two independent runs that were processed for 100 million generations. Tree sampling was performed each 100 generations, until the likelihood values of the two runs were stabilized with an average standard deviation of split frequencies at 0.011, and the potential scale-reduction factor (PSRF) of all parameters reached a value of 1.00. Burn-in of initial 25% generations allowed stabilization of the likelihood value in the set of sampled trees, and a 50% majority-rule consensus tree with estimated posterior probabilities of branches was constructed. The resulting tree was imported into Mesquite v. 3.51 (Maddison and Maddison, 2018) and a character matrix was created, in which the type of thylakoid arrangement best characterizing each strain in the data set was coded as categorical data (0 = absent, 1 = parietal, 2 = radial, 3 = fascicular, 4 = parallel, 5 = irregular, 6 = *Cyanothece* type, ? = unknown or equivocal). The thylakoid patterns were traced onto the phylogenetic tree using parsimony reconstruction of ancestral states (Parsimony Ancestral States option). The resulting tree was visualized using Inkscape 0.91 (available from www.inkscape.org) vector image editor. The CIPRES (Miller et al., 2012) supercomputing facility was used for calculation of the BI tree.

### Phylogenetic Analysis of Housekeeping Loci

A representative set of 136 cyanobacterial genomes supplemented with 12 outgroup bacterial genomes were used for analysis (Supplementary Table S2). Data for multilocus alignment were mined from a custom BLAST database compiled from the 148 genomes. Protein homologs were harvested by TBLASTN searches with an *E*-value of 1e^-10^ performed using BLAST+ 2.2.28 (Camacho et al., 2009). *Synechococcus lividus* PCC 6715 (NZ_CP01809) was selected as a reference organism due to its well-annotated genome and intermediate phylogenetic position among cyanobacteria. Amino acid sequences of all of its annotated proteins were BLASTed against the constructed BLAST database. The obtained 2548 sets of amino acid sequences were aligned and reordered using MAFFT with – localpair – reorder – maxiterate 1000 settings (Katoh and Standley, 2013). Whenever multiple BLAST hits for a single locus were retrieved from a particular genome, only the most similar hit was kept for subsequent analysis. Obtained alignments were further filtered so that only those containing more than 120 cyanobacterial sequences were kept for phylogenetic analysis. The resulting 260 alignments were considered to represent ubiquitous housekeeping loci (Supplementary Table S3) and they were concatenated providing a 313,388 amino acid alignment. Custom PERL scripts were applied for the BLAST hit filtering and the concatenation of alignments. The validity of the 260 selected protein alignments was tested using OD-seq (Jehl et al., 2015) and Guidance v. 2.02 (Sela et al., 2015) detecting no sequence outliers. All positions in the alignment with less than 80% site coverage were eliminated, providing a total of 156,762 sites in the final dataset. The tree inference was performed using the maximum-likelihood method with cpREV amino acid replacement model and 500 bootstrap (BP) repetitions (due to exceptional time requirements for analysis of such a long alignment) in MEGA 10.0.4 (Kumar et al., 2018).

### Auxiliary Analysis of Selected Proteins

Selected proteins with previously proposed effects on thylakoid or chloroplast morphology, and major protein components of thylakoid membranes, for which homologs could be identified in cyanobacterial genomes, were tested for their phylogenetic signal (Supplementary Table S4). The analyzed set included namely Vipp1 (Zhang et al., 2014), TerC (Kwon and Cho, 2008), CurT (Heinz et al., 2016), YidC/Alb3 (Sundberg et al., 1997), Riq1 and Riq2 (Yokoyama et al., 2016), and the prohibitin-like protein slr1768 (Bryan et al., 2011). Additionally, proteins involved in membrane phospholipid and glycolipid synthesis pathways, subunits of phycocyanin, allophycocyanin, and phycoerythrin; protein subunits of the photosystem I and II complexes; the cytochrome b6f; and the thylakoid ATP synthase were tested. Homologous proteins were harvested from our set of 136 representative cyanobacterial genomes as previously (the section “Phylogenetic Analysis of Housekeeping Loci”). In addition, all proteins except for the protein subunits employed in photosynthetic machinery were independently searched using the NCBI BLASTP suite ^1^ with common algorithm parameters but max target sequences increased to 500. The same procedure was repeated with Organism parameter set to Cyanobacteria (taxid:1117). Retrieved sequences were aligned and reordered using MAFFT with – localpair – reorder – maxiterate 1000 settings (Katoh and Standley, 2013). Initial phylogenetic trees from all protein alignments were calculated in FastTreeMP (Price et al., 2010) with – lg – gamma settings. Acquired phylogenetic trees were visually checked. In instances where they contained an unusual topology congruent with the distribution of thylakoid arrangements (e.g., separating all simple parietal type of thylakoids from the derived types), the phylogenetic trees were recalculated in MEGA 10.0.4 using maximum likelihood under LG+I+G model with 1000 *BP* repetitions.

## Results

### Thylakoid Arrangement and Cell Dimensions

**FIGURE 1:**
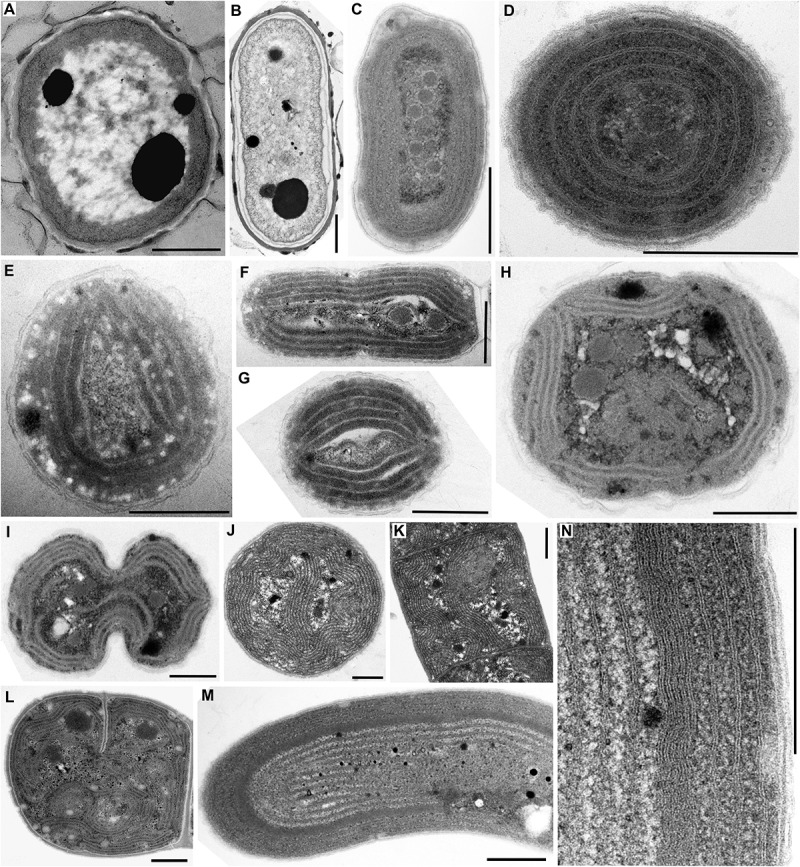
TEM documentation of absent thylakoids **(A,B)**, parietal thylakoid arrangement **(C,D)**, and its deviations **(E–N)**. Thylakoids were absent in **(A)** *Gloeobacter violaceus* PCC 6501 and **(B)** *G. violaceus* CCALA 981. **(C,D)** Plain parietal thylakoids in *Cyanobium gracile* PCC 6307; **(E–G)** two or three peripheral fascicles of thylakoids in *Leptolyngbya* sp. PCC 7376; **(H,I)** multiple fascicles of parietal thylakoids in *Synechocystis* sp. PCC 6714; **(J,K)** parietal arrangement with a central thylakoid fascicle in *Kamptonema animale* CCALA 771; **(L)** parietal arrangement with spherical formations in *Crinalium magnum* SAG 34.87; **(M,N)** concentric thylakoids with a dense subperipheral layer in *S. major* PCC 6313. Scale bars = 500 nm.

**FIGURE 2:**
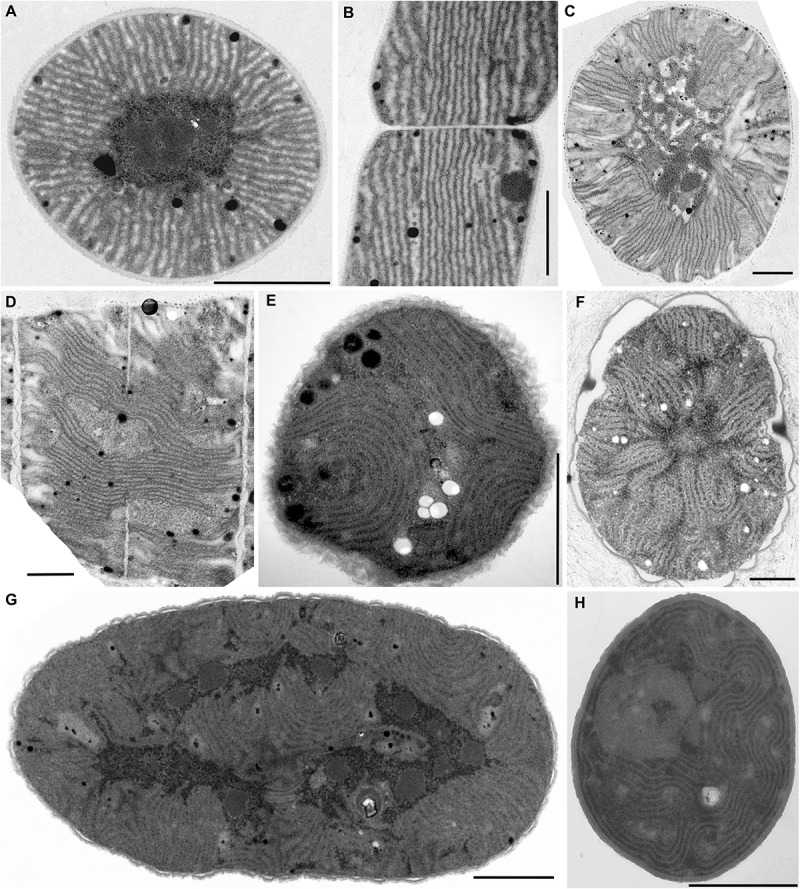
TEM documentation of radial **(A–D)** and fascicular **(E–H)** thylakoid arrangement. **(A,B)** Straight radial thylakoids in *Coleofasciculus chthonoplastes* PCC 7420; **(C,D)** flexuous radial thylakoids in *Arthrospira maxima* SAG 84.79; plain fascicular thylakoids in **(E)** *Cyanothece* sp. PCC 7424, **(F)** *Cyanothece* sp. PCC 7822, **(G)** *Cyanothece* sp. PCC 7424, and **(H)** *Chroococcidiopsis thermalis* PCC 7203. Scale bars = 1 μm.

**FIGURE 3:**
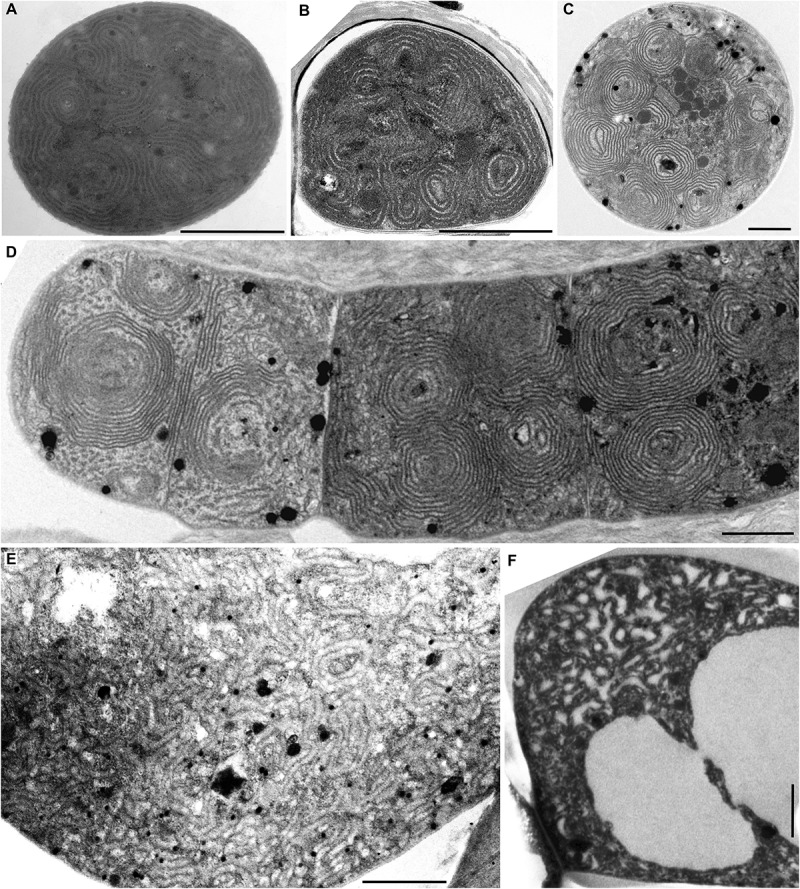
TEM documentation of fascicular thylakoid arrangement with prominent spherical formations **(A–D)**, and the irregular arrangement **(E,F)**. Fascicular and spherical formations in **(A)** *Synechocystis* sp. PCC 7509 and **(B)** *C. thermalis* PCC 7203. **(C,D)** Exclusively spherical formations in *Arthrospira* sp. PCC 8005. **(E,F)** Irregularly coiled thylakoids in *Stigonema ocellatum* SAG 48.90. Scale bars = 1 μm.

**FIGURE 4:**
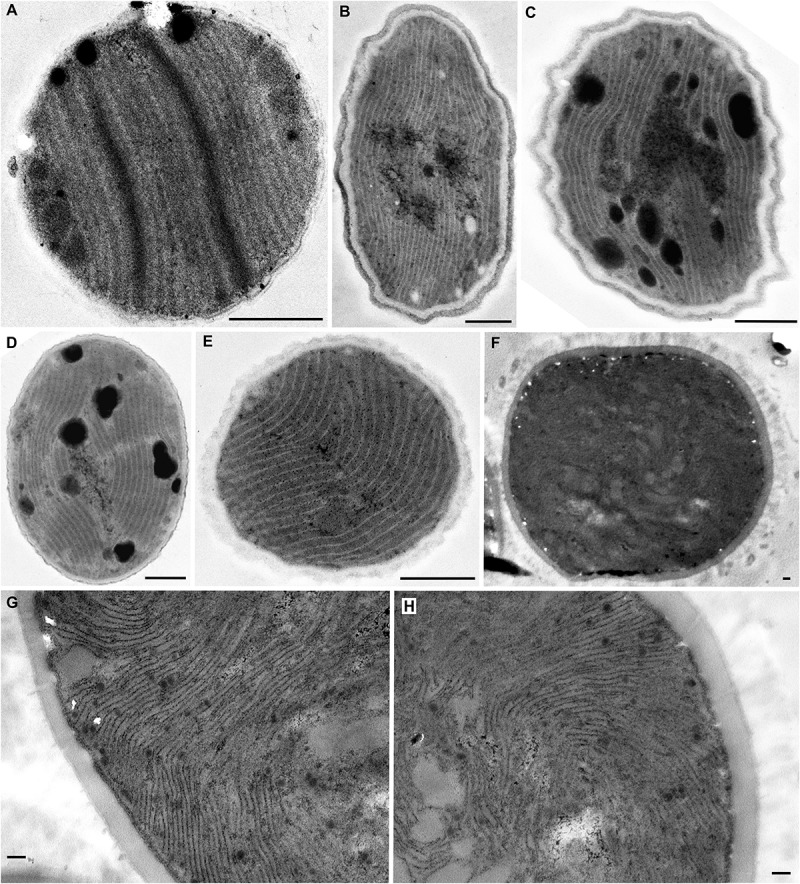
TEM documentation of parallel **(A–E)** and *Cyanothece*-like **(F–H)** thylakoids. A single array of parallel thylakoids filling the entire cell is visible in **(A)** *Cyanobacterium stanieri* PCC 7202 and **(B–E)** *Geminocystis papuanica* PAP1. **(F–H)** Thylakoids perpendicular to cell wall protrude into the cell center to form fascicular reticulate structures in *Cyanothece aeruginosa* SAG 87.79. Scale bars = 500 nm.

**FIGURE 5:**
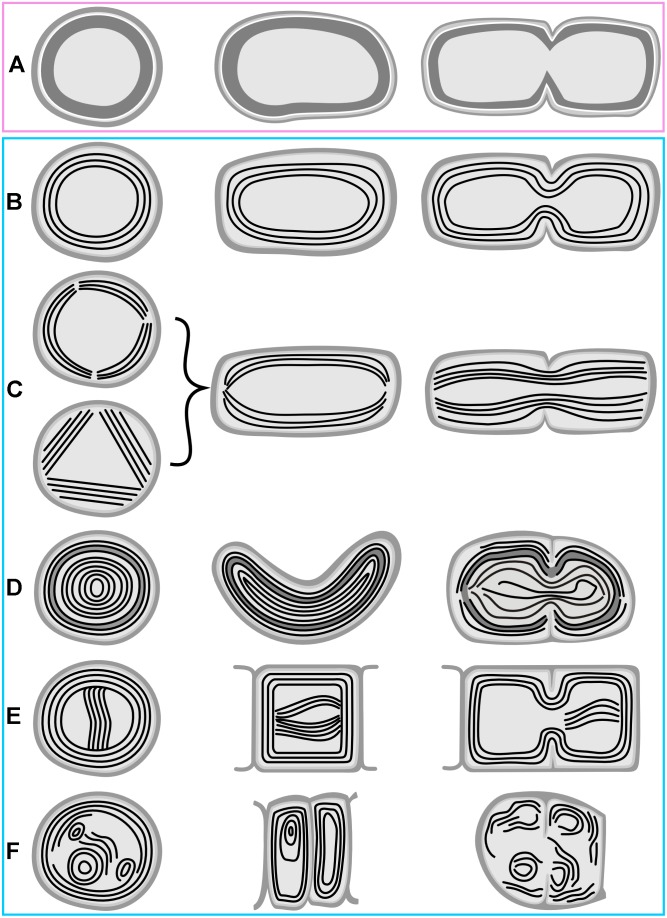
Schematic representation of basal types of thylakoid arrangement. **(A)** Thylakoid-less cells with a cortical layer of photosynthetic pigments; **(B)** simple parietal arrangement; **(C)** parietal thylakoids composed of peripheral fascicles; **(D)** parietal thylakoids with a subperipheral dense layer; **(E)** parietal thylakoids with a central fascicle; **(F)** parietal thylakoids with spherical formations. First column – cross section; second column – longitudinal section; third column – cell division. Panels are in boxes color-coded using the same coding as in Figure 8, 9.

The occurrence of all the main types and special features of thylakoid arrangement in cyanobacteria were documented by our TEM analysis of freshly grown strains (Figure 1–4). With few exceptions, almost all TEM data collected from literature could be fitted into categories listed in Supplementary Table S1, and the categories were subsequently used as a grouping variable in statistical analyses of cell dimensions and phylogenetic ancestral state reconstructions. A schematic representation of each thylakoid arrangement type and its special forms (where applicable) based on all available TEM data is provided in Figure 5, 6. A brief description of individual categories using our observations of ultrastructure follows.

**FIGURE 6:**
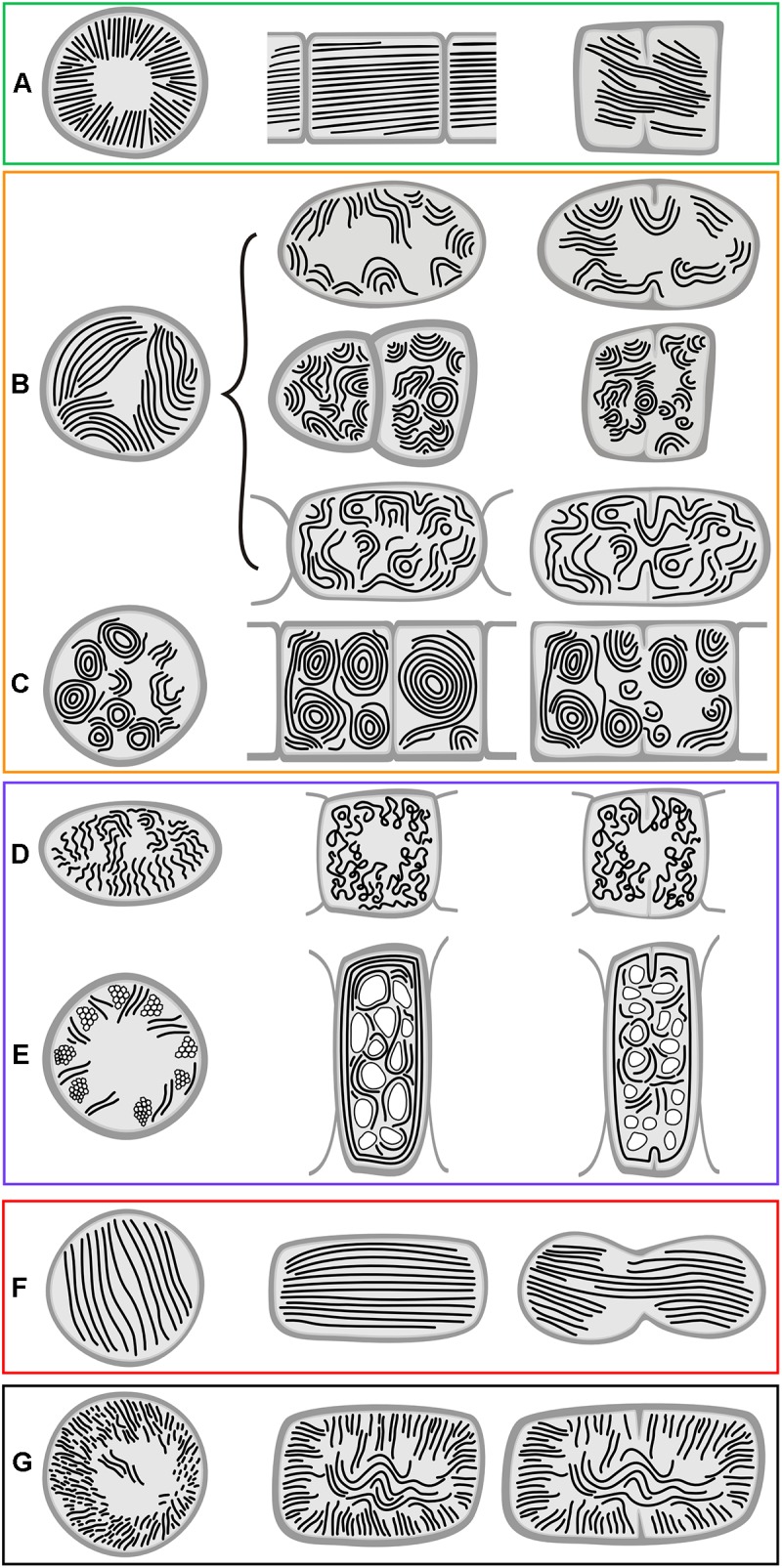
Schematic representation of derived types of thylakoid arrangement. **(A)** Radial arrangement; **(B)** fascicular arrangement and its modifications; **(C)** fascicular type with dominant spherical formations; **(D)** irregular arrangement in heterocytous cyanobacteria; **(E)** irregularly distributed thylakoids due to displacement by cellular inclusions; **(F)** parallel arrangement; **(G)** *Cyanothece*-like arrangement. First column – cross section; second column – longitudinal section; third column – cell division. Panels are in boxes color-coded using the same coding as in Figure 8, 9.

#### Absence of Thylakoids

A typical fine structure of cells, lacking thylakoids but containing a cortical electron-dense layer and prominent polyphosphate granules, was found in the strains of unicellular cyanobacterium *Gloeobacter violaceus*, PCC 6501 and CCALA 981 (Figure 1A,B, 5A).

#### Parietal Arrangement of Thylakoids

A simple parietal arrangement with several concentric, uninterrupted layers of thylakoid membranes (Figure 5B) was observed in the coccal picocyanobacterium *Cyanobium gracile* PCC 6307 (Figure 1C,D). In some strains, parietal thylakoids frequently formed two to several fascicles lining the cell periphery (Figure 5C) as documented in both filamentous (*Leptolyngbya* sp. PCC 7376, Figure 1E,F) and coccal (*Synechocystis* sp. PCC 6714, Figure 1H,I) strains. In PCC 7376, three such fascicles were commonly present, resulting in a triangular thylakoid arrangement observed in the cross-section (Figure 1E). Another modification to the typical parietal arrangement was exemplified in the filamentous strain *Kamptonema animale* CCALA 771, in which a fascicle of thylakoid membranes sometimes protruded into the center of the cell, in addition to the thylakoids lining the cell wall (Figure 1J,K, 5E). Rarely, strains exhibiting a prevailably parietal architecture formed distinct spherical formations in their cells (Figure 5F) as documented in the strain *Crinalium magnum* SAG 34.87 (Figure 1L). A new special feature was established by TEM documentation of *Spirulina major* PCC 6313 (Figure 1M,N), in which the thylakoids were organized concentrically, filling almost the entire cell, frequently showing a subperipheral layer of tightly stacked membranes (Figure 1N, 5D).

#### Radial Arrangement of Thylakoids

Another type of thylakoid architecture (Figure 2A–D) was shown in two strains of filamentous cyanobacteria (*Coleofasciculus chthonoplastes* PCC 7420, *Arthrospira maxima* SAG 84.79). The thylakoids were organized radially (Figure 6A), perpendicular to the cell wall in the cross-section (Figure 2A,C), appearing as lengthwise parallel lines in the longitudinal section (Figure 2B,D).

#### Fascicular Arrangement of Thylakoids

A different structural pattern, particularly found in representatives of derived coccoid, filamentous, and heterocytous cyanobacteria, was verified in “*Cyanothece*” strains PCC 7424 and PCC 7822, *Chroococcidiopsis thermalis* PCC 7203, “*Synechocystis*” sp. PCC 7509, and *Arthrospira* sp. PCC 8005 (Figure 2E–H, 3A–D). These strains exhibited thylakoids arranged in variously coiled and wavy fascicles of membranes, forming short parallel segments or hemispherical loops (Figure 2E,H, 6B). In some cases, the loops reached fully spherical formations (Figure 6C), very abundant and prominent especially in the strain *Arthrospira* sp. PCC 8005 (Figure 3C,D).

#### Irregular Arrangement of Thylakoids

Another category can be distinguished from the fascicular type. The thylakoids of this type also appear irregularly coiled, but do not form (or only rarely) distinct fascicles composed of several parallel membranes (Figure 6D). This was documented by the analysis of *Stigonema ocellatum* SAG 48.90 (Figure 3E,F). Disordered distribution was also found in cyanobacteria with extensive vacuolization of the cells (Supplementary Table S1 and Figure 6E).

#### Parallel Arrangement of Thylakoids

The following category of thylakoid arrangement seems to be quite unique. It was found in the coccoid genera *Cyanobacterium* (*C. stanieri* PCC 7202) and *Geminocystis* (*G. papuanica* PAP1) in which thylakoids appeared as parallel lamellae filling the whole cell in both cross and longitudinal sections (Figure 4A–E, 6F). The longitudinal section resembled that of the radial type (Figure 2B), however no radial formations were found in the cross-section.

#### *Cyanothece* Type of Thylakoids

The last category seems to be thus far exclusive for *Cyanothece sensu stricto* (Figure 4F,H). Examination of strain *Cyanothece aeruginosa* SAG 87.79 demonstrated some resemblance to the radial type, especially in peripheral regions of the large cell (Figure 4G,H), however we split it from that category. The thylakoids in *C. aeruginosa* were not merely radial, the cell contained also fascicles that continued into its center, forming an irregularly coiled net (Figure 4F, 6G).

#### Morphometric Analysis

**FIGURE 7:**
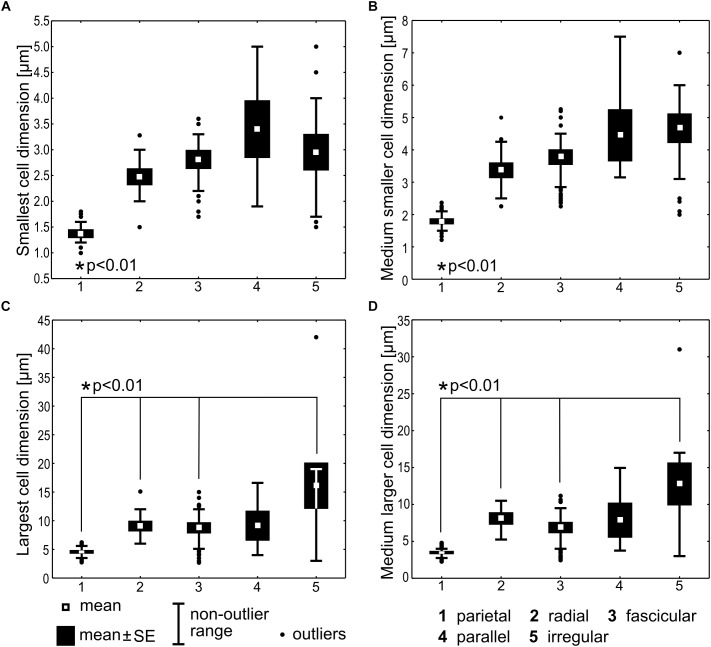
Statistical comparison of cell dimensions across the types of thylakoid arrangement. The smallest cell dimension **(A)** represents the lower extreme of width (cylindrical cells) or length (discoid cells) interval for the particular strain. The medium smaller cell dimension **(B)** represents the middle value of this interval. The largest **(C)** and the medium larger **(D)** cell dimensions are analogous values (the upper extreme and the middle value, respectively) of the bigger cell diameter. Kruskal–Wallis test (*N* = 204, *p* < 0.001) was applied for testing the variance among thylakoid arrangement categories and two-tailed *t*-tests (*p* < 0.01) were used for pair-wise comparisons. Statistically significant differences are marked with asterisks.

Statistical comparison of cell dimensions was performed among the categories of thylakoid arrangement, excluding the *Cyanothece* type represented by a single strain, and *Gloeobacter* lacking the thylakoids. Significant variability (Kruskal–Wallis test, *N* = 204, *p* < 0.001) was detected in the data sets for each of the analyzed morphometric variables: the smallest cell dimension, the largest cell dimension, the medium smaller cell dimension, and the medium larger cell dimension (Figure 7). Specifically, the smallest and the medium smaller cell dimensions in the parietal type were significantly lower than in each of the remaining types (*t*-test, *p* < 0.01). The largest and the medium larger cell dimensions were also significantly lower (*t*-test, *p* < 0.01) in the parietal type than in the radial, fascicular, and irregular types, but did not significantly differ from the parallel type. Apart from the parietal category, the remaining types of arrangements did not significantly differ in their respective cell dimensions.

The parietal type exhibited the average smallest cell diameter of 1.37 (±0.07) μm, and the average smaller cell dimension of 1.79 (±0.08), while the remaining types had cells in average not smaller than 2.48 (±0.14)–3.40 (±0.54) μm and their average smaller cell dimension ranged 3.37(±0.23)–4.67 (±0.43) μm. In cyanobacteria with cylindrical cells, the smaller cell dimension can be interpreted as cell width, and the larger one as cell length. Therefore, we can summarize that typical cyanobacteria with parietal thylakoids had cells in average less than 1.8 μm wide, which distinguished them in a statistically significant way from cyanobacteria with non-parietal thylakoids, having cells in average not less than 2.5–3.4 μm wide, but usually 3.4–4.7 μm wide.

### SSU rRNA Gene Tree

**FIGURE 8:**
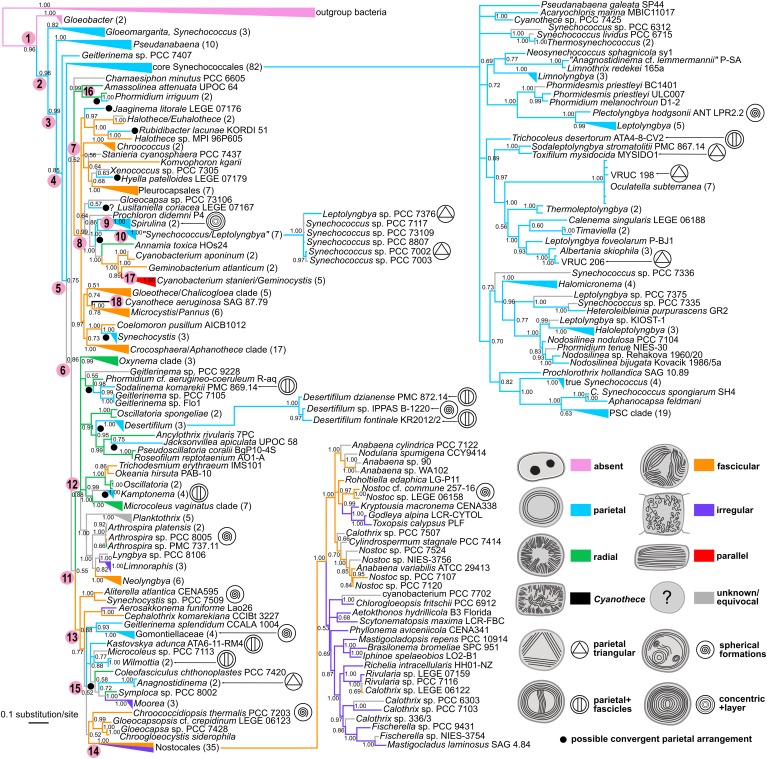
Phylogenetic tree of cyanobacteria inferred from the SSU rRNA gene with traced evolution of thylakoid arrangement. The tree was calculated using BI from an alignment of 298 sequences spanning 1477 nucleotide positions. Estimated posterior probabilities are displayed near the nodes. Numbers in parentheses represent the number of sequences in collapsed branches. Color-coded thylakoid arrangements in non-terminal nodes were inferred using the parsimony ancestral reconstruction method. Special features in thylakoid architecture are indicated with pictograms. Reconstructed convergent occurrences of the parietal arrangement are marked with filled black circles. Selected nodes, which are referred to in the main text, are numbered to facilitate tree description. Collapsed nodes containing taxa with a single category of thylakoid arrangement together with taxa with unknown ultrastructure are color-coded according to the known category. PSC clade = *Prochlorococcus*, marine *Synechococcus*, and *Cyanobium*.

The phylogenetic reconstruction yielded a tree (Figure 8), which, after rooting with outgroup bacteria, showed *Gloeobacter* as the basal lineage with 0.96 posterior probability (PP). The basal lineage (node 1 in Figure 8) was immediately followed by several paraphyletic clades of synechococcalean cyanobacteria (nodes 2–5) such as the coccoid genera *Synechococcus*, *Acaryochloris*, *Thermosynechococcus*, the marine picocyanobacteria (*Prochlorococcus*, marine *Synechococcus*, and *Cyanobium*, abbreviated as PSC), and the simple filamentous types such as *Pseudanabaena*, *Leptolyngbya*, *Phormidesmis*, *Nodosilinea*, *Halomicronema*, etc. The deep branching of these clades was relatively well supported (0.75–0.99 PP).

Two more derived large lineages arose from the following node (node 6). First of them (node 7) received only a 0.52 PP, and contained a subclade (node 8) of prevailably chroococcalean and pleurocapsalean cyanobacteria, but also *Rubidibacter*, *Prochloron*, *Jaaginema litorale*, the *Spirulina* cluster (node 9), and a group of six strains (node 10) designated as *Synechococcus* and *Leptolyngbya* (0.99 PP). Several strains (*Chamaesiphon minutus*, *Amassolinea attenuata*, and *Phormidium irriguum*) were resolved in a position basal to this subclade.

The second lineage (node 11) again immediately split. One of the descendent clades (node 12; 0.88 PP) included exclusively oscillatorialean cyanobacteria such as *Oscillatoria*, *Phormidium*, *Microcoleus*, *Kamptonema*, *Planktothrix*, *Arthrospira*, *Oxynema*, etc. The second descendent clade (node 13; 0.88 PP) was more heterogeneous and showed a less resolved internal structure. It harbored several types of coccoid cyanobacteria such as *Aliterella*, *Chroogloeocystis*, and single strains of polyphyletic morphogenera *Chroococcidiopsis*, *Gloeocapsa*, *Gloeocapsopsis*, and *Synechocystis*. It further contained several characteristic oscillatorialean cyanobacteria from the family *Gomontiellaceae*, *Geitlerinema sensu stricto*, single strains of *Microcoleus* and *Symploca*, and several recently erected genera (*Aerosakkonema*, *Cephalothrix*, *Kastovskya*, *Wilmottia*, *Coleofasciculus*, and *Moorea*). The last member of the clade was a fully supported cluster (node 14) of 35 heterocytous cyanobacteria – Nostocales (1.00 PP).

Ancestral state reconstruction analysis revealed partial congruence of the designated thylakoid arrangement categories with the SSU rRNA phylogeny (Figure 8). The single thylakoid-less cyanobacterial genus *Gloeobacter* occupied the base of the tree (node 1). The several following synechococcalean clades (nodes 2–5, 96 sequences in total) unequivocally exhibited the parietal arrangement of thylakoids, which was therefore reconstructed as plesiomorphic in thylakoid-bearing cyanobacteria.

The parietal type was however found to exhibit extensive homoplasy. At least 10 convergent occurrences of the parietal arrangement were suggested by ancestral state reconstruction using the SSU rRNA tree. Typical examples are represented by the “*Synechococcus*/*Leptolyngbya*” clade (node 10), *Synechocystis*, and *Rubidibacter* clustering within the prevailably chroococcalean lineage (node 8), and by *Geitlerinema*, *Kastovskya*, and *Anagnostidinema* occupying one of the most derived lineages (node 15) leading toward the Nostocales. On the other hand, the *Spirulina* cluster (node 9), which was also resolved among derived coccoid cyanobacteria (node 8), showed a unique sub-peripheral layer of dense thylakoid membranes that could distinguish it from the parietal category (Figure 1M,N, 5D).

Two types of thylakoid arrangement emerged more or less simultaneously from the ancestral parietal architecture based on our SSU rRNA reconstruction. First of them, the radial type, was reconstructed as ancestral in the lineage (node 12) consisting exclusively of Oscillatoriales (derived unbranched filamentous cyanobacteria without heterocytes). It further occurred in a clade consisting of *Amassolinea* and *P. irriguum*, weakly clustering as a clade basal to the chroococcalean/pleurocapsalean lineage (node 16; 0.52 PP). Thylakoid arrangements highly convergent to the radial type were found in three strains tightly anchored in clades isolated from the main “radial lineage” – *Coleofasciculcus chthonoplastes* PCC 7420, *Symploca* sp. PCC 8002, and *Annamia toxica* Hos24.

The second type was the fascicular arrangement of thylakoids. Thylakoids falling to this category were identified as ancestral in the main chroococcalean/pleurocapsalean lineage (node 8), as well as in the mixed lineage leading toward Nostocales (node 13). This arrangement was therefore typically found in phylogenetically derived coccoid cyanobacteria, and part of heterocytous cyanobacteria. Only rarely it occurred in filamentous types such as *Trichodesmium* and *Okeania* (sister taxa), *Neolyngbya*, and *Cephalothrix*.

An irregular thylakoid pattern, similar to the previous fascicular one but lacking distinct fascicles, was typically found only in two subgroups of heterocytous cyanobacteria (node 14, Nostocales). Thylakoids were irregularly distributed also in two unrelated genera of thick filamentous cyanobacteria, *Limnoraphis* and *Moorea* (Figure 6E).

The parallel type of thylakoid architecture was exclusively found in a single clade (1.00 PP) comprising two genera, *Cyanobacterium* and *Geminocystis* (node 17). Finally, *C. aeruginosa* (node 18), which exhibits a special type of thylakoid arrangement (Figure 6G), was resolved in the main coccoid lineage as a sister taxon to the *Microcystis*/*Pannus* group (0.78 PP) in the SSU rRNA tree.

### Phylogenomic Tree

**FIGURE 9:**
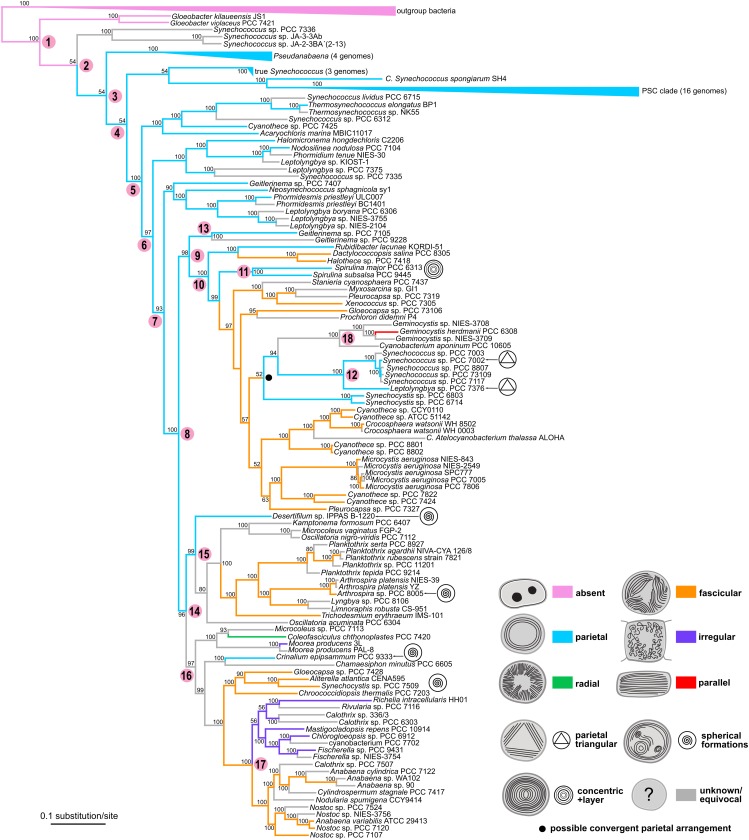
Phylogenetic tree of cyanobacteria inferred from 260 housekeeping proteins with traced evolution of thylakoid arrangement. The tree was calculated using the maximum-likelihood method from an alignment of 148 sequences rows spanning 156,762 amino acid positions. BP values (500 repetitions) are displayed near the nodes. Numbers in parentheses represent the number of sequences in collapsed branches. Color-coded thylakoid arrangements in non-terminal nodes were inferred using the parsimony ancestral reconstruction method. Special features in thylakoid architecture are indicated with pictograms. Reconstructed convergent occurrence of the parietal arrangement is marked with a filled black circle. Selected nodes, which are referred to in the main text, are numbered to facilitate tree description. Collapsed nodes containing taxa with a single category of thylakoid arrangement together with taxa with unknown ultrastructure are color-coded according to the known category. PSC clade = *Prochlorococcus*, marine *Synechococcus*, and *Cyanobium*.

The multilocus tree based on 260 proteins (Figure 9) was in many respects congruent with the SSU rRNA tree (Figure 8). When rooted with outgroup bacteria, it contained the basal *Gloeobacter* lineage (node 1 in Figure 9) supported by a 100% BP value. It was followed by several paraphyletic synechococcalean clades (nodes 2–7, 54–100% BP) harboring a similar set of taxa as in the SSU rRNA tree (*Synechococcus*; marine picocyanobacteria *Prochlorococcus*, marine *Synechococcus*, and *Cyanobium* abbreviated as PSC; *Pseudanabaena*; *Leptolyngbya*, etc.).

The following node (node 8, 100% BP) gave rise to two large groups of derived cyanobacteria. The first of them (node 9, 96% BP) included a well-supported lineage (node 10, 100% BP) highly resembling node 8 in the SSU rRNA tree, as it was composed of a mixture of chroococcalean and pleurocapsalean cyanobacteria, *Prochloron*, *Rubidibacter*, the *Spirulina* clade (node 11, 100% BP), and the “*Synechococcus*/*Leptolyngbya*” clade (node 12, 100% BP). Two strains of *Geitlerinema sensu lato* (node 13) were resolved in a position basal to node 10. This contradicted the topology based on the SSU rRNA gene, in which these strains were found within the main oscillatorialean clade.

The second large lineage (node 14, 96% BP) split into two subclades that were again highly identical to those recovered in the SSU rRNA tree (nodes 9 and 10 in Figure 8). One of them (node 15, 99% BP) contained exclusively oscillatorialean cyanobacteria, while the second one (node 16, 97% BP) was a mixed clade of coccoid and filamentous types, including the distinct lineage of heterocytous cyanobacteria (node 17, 100% BP).

Besides the two strains of *Geitlerinema sensu lato* mentioned earlier, the two phylogenetic trees exhibited further minor incongruences, mostly in clustering of several isolated strains. It concerned especially *C. minutus* PCC 6605, which formed an unresolved branch close to the main coccoid lineage in the SSU rRNA tree, whereas in the multilocus tree it was a sister branch of *Crinalium epipsammum* PCC 9333. Furthermore, *Spirulina* and *Synechocystis* occupied variable positions in the main coccoid lineage when comparing between the two trees.

The ancestral state reconstruction analysis using the multilocus phylogeny (Figure 9) corroborated the primordial absence of thylakoids in *Gloeobacter* (node 1) and plesiomorphic character of the parietal arrangement (nodes 2–8). In contrast to the SSU rRNA tree, the multilocus tree suggested only rare homoplasy of the parietal arrangement. Strains exhibiting this thylakoid arrangement were on several occasions resolved in basal positions within their clades in the multilocus tree, which supported possible ancestral occurrence of parietal thylakoids in these groups. Specifically, two *Geitlerinema* strains (node 13), *Rubidibacter*, and *Spirulina* clustered on the basis of the main coccoid lineage (nodes 9–10), *Desertifilum* was basal in the main oscillatorialean group (node 15), and *Crinalium* fell into one of the basal branches in the lineage (node 16) leading toward Nostocales. Only a single convergence event reverting the fascicular architecture back to the parietal one was unequivocally supported in the multilocus tree, which involved *Synechocystis* and the “*Synechococcus*/*Leptolyngbya*” cluster (node 12) embedded in the main coccoid lineage (node 10).

In the evolutionary scenario suggested by the multilocus analysis, the fascicular arrangement of thylakoids evolved independently two to three times from the parietal arrangement. Independent origin of the fascicular type in the main coccoid lineage (node 10), the mixed lineage (node 16) leading toward Nostocales and part of the core oscillatorialean clade (node 15) did not contradict the SSU rRNA-based reconstruction. The occurrence of the irregular thylakoid architecture derived from the fascicular one was again analogous in both trees.

A slightly different phylogenetic pattern between the two trees was identified in the radial category. Both trees suggested a probable independent origin of this arrangement in the *C. chthonoplastes* lineage. Due to lack of strains with both WGS and TEM data in the main oscillatorialean lineage (node 15), the multilocus tree did not provide evidence on the radial arrangement in this clade. Nevertheless, as noted earlier, the ancestor of this cluster was (at least for now) reconstructed to have had parietal not radial thylakoids.

Finally, the parallel arrangement of thylakoids, found in the *Cyanobacterium*/*Geminocystis* lineage (node 18), was found in corresponding phylogenetic positions in both trees. However, in the SSU rRNA tree it was reconstructed to have evolved from the fascicular type, while in the multilocus tree it could have originated from the convergent parietal type acquired by the ancestor of *Synechocystis* and node 12. Origin of the thylakoid structure typical for *Cyanothece sensu stricto* was not addressed in the multilocus tree due to missing genome sequencing data.

### Phylogenetic Patterns of Selected Proteins

Phylogenetic trees based on photosynthetic and proposed thylakoid-forming proteins usually showed evolutionary patterns slightly different to phylogenies derived from the SSU rRNA gene and the 260 housekeeping proteins (Mareš et al., 2018).

Vipp1 search confirmed its similarity to phage shock protein PspA in *Escherichia coli*. Its phylogeny retained roughly the same clades of cyanobacteria as in the genome-wide tree, with exception of *Synechococcus* sp. PCC 7336 which was in a position basal to *Gloeobacter*, and *Crinalium* and *Rubidibacter* (long branches) clustering in different clades. Photosystem I assembly protein BtpA provided a peculiar phylogeny, although unrelated to thylakoid morphology. BtpA from *Chloroflexus* was basal to marine *Synechococcus*, followed by *Gloeobacter*. It was not found in *Prochlorococcus*, otherwise it corresponded to the phylogenomic tree. On the other hand, YidC/Alb3 phylogeny was generally congruent with the trees derived from housekeeping loci.

CurT was initially not recovered from 42 genomes in our genomic database, being too diverse to provide hits under the selected search settings. In a dedicated BLASTP search across the NCBI database (including the conserved domain database search), it was clearly missing in *Gloeobacter* and in a basal group of thermal *Synechococcus* from Yellowstone and *Gloeomargarita*. It provided doubtful hits (<50% coverage, around 20–30% identity to CurT from *Synechocystis*) in picocyanobacteria *Prochlorococcus*, (marine) *Synechococcus*, and *Cyanobium*. TerC was not recovered from majority of cyanobacterial genomes both in our genomic database and in GenBank. Riq1 and Riq2 were completely missing in cyanobacteria.

Phylogenies of proteins related to photosynthesis, proteins of membrane phospholipid and glycolipid biosynthesis pathways, and subunits of phycocyanin, allophycocyanin, and phycoerythrin showed various types of phylogenetic relationships. The basal cyanobacterial groups and other groups that clustered on the level of cyanobacterial genera and families remained phylogenetically close, but their positions were intermixed among the trees. In almost every phylogenetic tree of a particular protein exceptions in phylogenetic placement of several strains different from topology of the SSU rRNA or genomic tree were found, however, these differences did not exhibit any specific relation to thylakoid morphology.

In summary, none of the trees showed significant congruences with the designated thylakoid arrangement categories (additional to those reflected in the general phylogeny of concatenated housekeeping loci). Some of the proteins with postulated roles in thylakoid biogenesis and morphology were missing from certain or all translated cyanobacterial genomes. Due to lack in informativeness we do not show the trees in the article, but they can be accessed for comparison in the Figshare web repository (Mareš et al., 2018).

## Discussion

**FIGURE 10:**
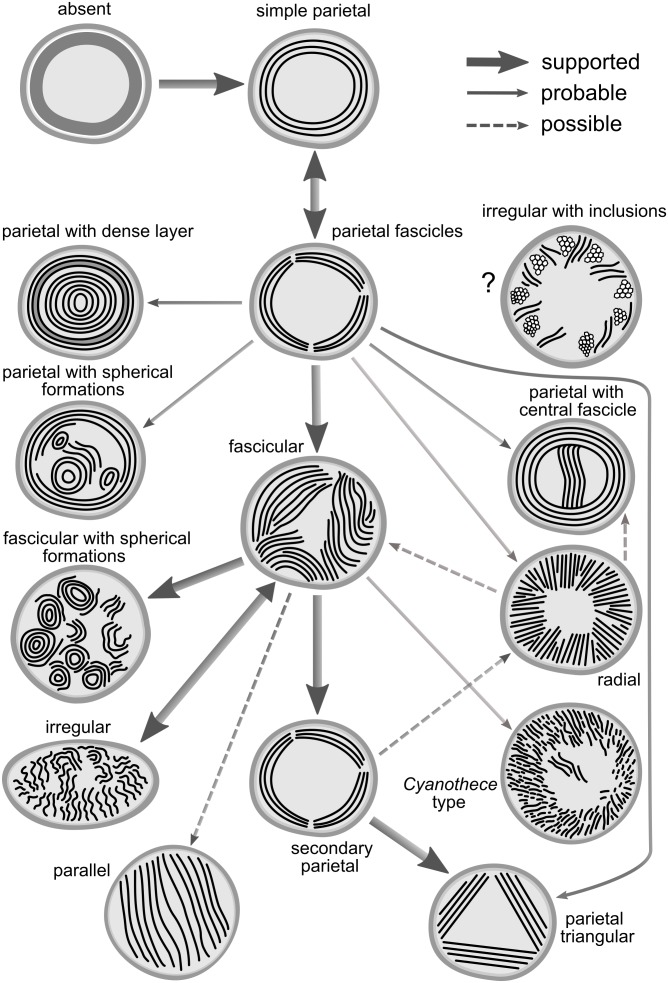
Hypothetical concept of evolutionary routes connecting the known thylakoid architectures. Thick solid lines indicate relationships supported in both the SSU rRNA and the multilocus tree. Fine solid lines indicate probable relationships inferred from one of the trees but missing (due to taxon sampling) or equivocal in the second tree. Dashed lines indicate possible relationships with low support or contradictory between the two trees.

Synthesis of phylogenetic analysis based on a well-sampled SSU rRNA database (Figure 8) and genome-wide phylogeny inferred from 260 conserved loci (Figure 9) resulted in a relatively congruent picture of the evolution of subcellular thylakoid architecture (Figure 10). Ancestral state reconstruction analyses suggested its partial determination by inheritance and frequent conservation in individual lineages in agreement with previous studies (Komárek and Kaštovský, 2003). The practical use of thylakoid architecture in taxonomy is however strongly limited due to both plesiomorphy and homoplasy. Correlation of thylakoid arrangement with photosynthetic membrane proteins and putative thylakoid-forming elements did not reveal any clear phylogenetic patterns, but provided evidence that some of the proteins postulated in thylakoid biogenesis are unlikely to be essential in all species.

### Evolution of the Thylakoid Membrane System

The closest common ancestor of extant cyanobacteria could have lacked thylakoids. This is in agreement with the notoriously reported basal position of *Gloeobacter*, the single known thylakoid-less cyanobacterium (Rippka et al., 1974; Guglielmi et al., 1981; Mareš et al., 2013). Nevertheless, we emphasize that a secondary loss of thylakoids in *Gloeobacter* is equally possible from the probabilistic point of view – thylakoids either emerged once or were once lost in the evolution of known cyanobacteria.

Whether primary or secondary, the ancestral fine structure in thylakoid-bearing cyanobacteria involves a parietal arrangement (Figure 1, 5). Peripheral thylakoids lining the inner surface of the cell walls are typically found in single-celled and filamentous strains occupying the paraphyletic basal branches (Komárek et al., 2014; Figure 8, 9). As an example, analysis using cryo-electron tomography suggested thylakoid membranes forming nearly spherical concentrical lamellae interconnected by distinct junctions in *Prochlorococcus* (Ting et al., 2007).

According to our results, a parietal-like arrangement has likely been introduced into derived clades by convergence – once or twice according to the multilocus phylogeny (Figure 9), serially according to the SSU rRNA gene phylogeny (Figure 8). Serial convergence creating plesiomorphic (*Synechococcus*-like) morphotypes in cyanobacteria was previously noted by Dvořák et al. (2014), although ultrastructure was not analyzed in that study. Three-dimensional structure of the convergent type of parietal arrangement has been addressed in experimental strains *Synechocystis* sp. PCC 6803 (Liberton et al., 2006; van de Meene et al., 2006) and *Synechococcus* sp. PCC 7002 (Nierzwicki-Bauer et al., 1983). In both cases it was composed of several interconnected fascicles of lamellar sheets lining the cell wall. Such an architecture however does not seem to be completely unique for the convergent morphotypes. Parietal lamellae split into two to three fascicles have been reported also in the basal clades (Figure 8), especially in filamentous cyanobateria such as *Pseudanabaena* (Guglielmi and Cohen-Bazire, 1984), *Phormidesmis* (Komárek et al., 2009), *Oculatella* (Bruno et al., 2009), *Toxifilum* (Zimba et al., 2017), and others.

We noticed that cyanobacteria from the basal clades usually exhibited small cell dimensions (Supplementary Table S1). Therefore, we tested the possible correlation between cell dimensions and thylakoid arrangement. Our results demonstrate the smaller of cell diameters (width in cylindrical cells/length in discoid cells) to be significantly lower in cyanobacteria with parietal thylakoid arrangement compared to other categories (Figure 7). Remarkably, this applied also to cyanobacteria with an unambiguously secondary occurrence of the parietal arrangement (Figure 9), including *Synechocystis* sp. PCC 6803 and PCC 6714 (1.4–2.5 μm) and the convergent clade (node 12 in Figure 9) of *Synechococcus*/*Leptolyngbya* (1–1.5 μm in width).

We therefore speculate that cyanobacterial cells with the smallest diameter under approximately 2 μm present a physiological limitation to complex thylakoid architectures (other than the parietal one). It seems plausible that cell size determines the occurrence of this type of thylakoids in both basal and derived clades of cyanobacteria rather than genetic factors. It is not surprising that small cell size has evolved independently several times in cyanobacteria. It can serve as an adaptation strategy to environments with limited nutrients providing larger surface-to-volume ratios (Harris and Theriot, 2016) as well as lower sedimentation rates (Stokes’ law). Small cells with thylakoids displaced toward the cell wall can also shorten the diffusion path length of energy metabolites in the cell. The variation in cell and filament morphology in response to various environmental factors to maximize photosynthetic effectiveness is well documented in cyanobacteria (Montgomery, 2015).

In cyanobacteria (both unicellular and filamentous) with cells always larger than approximately 2.5 μm the most frequent thylakoid arrangement appears as irregularly coiled fascicles distributed throughout the whole cell, although often more dense near the cell periphery (Figure 2E–H, 6B). A three-dimensional model of this category was previously exemplified using electron tomography in *Cyanothece* sp. ATCC 51142, showing a dense network that extends throughout the entire cell, partly exhibiting a quasi-helical organization (Liberton et al., 2011a,b). To what extent this model can be generalized in cyanobacteria with fascicular thylakoids remains to be determined in future studies. The fascicular arrangement also seems to have evolved multiple times (Figure 8, 9).

In heterocytous cyanobacteria, irregularly coiled and entangled thylakoids (Figure 3E,F, 6D) were recorded in two sub-clades in our study. This organization seems to have evolved from the fascicular type however its exact three-dimensional model is unavailable at the moment. Thick filamentous cyanobacteria also occasionally exhibit irregularly distributed thylakoids (Figure 6E), which may however be partly due to content of gas vacuoles (aerotopes) or a high degree of cell compartmentalization (Engene et al., 2012; Komárek et al., 2013). Limited free space in their cells seems to restrict the formation of any organized thylakoid structures.

Several types of unbranched filamentous cyanobacteria have evolved a peculiar radial organization of thylakoids (Figure 2A–D, 6A). This arrangement makes an impression of a highly organized structure with a continuity of membrane orientation between neighboring cells (Lokmer, 2007; Rasoulouniriana et al., 2009; Chatchawan et al., 2012; Figure 2B,D). The three-dimensional structure has not been investigated, however comparison of cross and lengthwise sections suggests that thylakoids form longitudinal belts oriented in perpendicular planes to the cell wall. Surprisingly, this organization seems to have evolved independently at least three times (Figure 8). It is therefore tempting to speculate that the radial arrangement is somehow connected to relatively thin unbranched filaments (2–10 μm) or to yet unknown physiological adaptations of this type of cyanobacteria.

According to our reconstructions, a single evolutionary event led to the origin of parallel thylakoid arrangement, either from fascicular (Figure 8) or convergent parietal (Figure 9) ancestors. This architecture typical for few unicellular cyanobacteria (Rippka and Cohen-Bazire, 1983; Komárek et al., 2004; Korelusová et al., 2009) seems to involve a single longitudinal array of thylakoid lamellae filling the entire cell.

The last unique type of organization was thus far found exclusively in *Cyanothece sensu stricto* (Komárek and Cepák, 1998; Komárek et al., 2004; Figure 4F–H, 6G). It is similar to the radial type with thylakoids perpendicular to cell wall, however forming an irregular network in the cell center. The exact three-dimensional structure is unknown. This architecture emerged among other unicellular cyanobacteria in our SSU rRNA tree (Figure 8), which contradicts the previously reported position of *C. aeruginosa* close to filamentous cyanobacteria (Bohunická et al., 2015a). Its phylogenetic position requires future confirmation, ideally using whole-genome sequencing data.

The general evolutionary scheme of thylakoid arrangements (Figure 10) only partly matches the one proposed by Komárek and Kaštovský (2003). Both evolutionary hypotheses agree in the ancestral position of thylakoid absence, plesiomorphic character of parietal types, and the probable direct emergence of the irregular type from the fascicular architecture. On the other hand, single origin of the parietal arrangement, as well as speculation about evolutionary links between parallel or *Cynaothece*-like type and the radial type are clearly contradicted by our results.

### Taxonomic Value and Stability of Thylakoid Arrangement

Is the thylakoid architecture a good taxonomic character in cyanobacteria? The value of taxonomic traits in general recorded in these microbes is highly dependent on the particular taxonomic scale and resolution, which is interesting to us (Komárek, 2018). Based on our results we can conclude that thylakoid arrangements are too unstable and burdened by convergence to be used as a taxonomic character separating cyanobacterial orders, families, and, in some instances, even genera. Unfortunately, the situation resembles those reached with other phenotypic features such as multicellularity (Schirrmeister et al., 2011), true branching (Gugger and Hoffmann, 2004; Mareš et al., 2015), and heteropolarity (Shalygin et al., 2017). Our findings support the idea of serial convergent evolution of cyanobacterial phenotypes across their extremely long evolutionary history (Dvořák et al., 2014).

The absence of thylakoids is taxonomically informative, as it is exclusive for *Gloeobacter* (Gloeobacterales). On the other hand, parietal arrangement, unless split and re-defined in future, does not seem to be a useful general character due to both homoplasy and plesiomorphy. Plesiomorphy is suggested in the current study by occurrence in paraphyletic taxa of the derived lineages (Figure 9), while both plesiomorphy and homoplasy is repeatedly evidenced in the polyphyletic order Synechococcales (Komárek et al., 2014; Mareš et al., 2018, compare also to Synechococcophycidae in Hoffmann et al., 2005). The paraphyletic parietal thylakoid architecture in derived clades applies, e.g., to *Rubidibacter* (Choi et al., 2008), *Spirulina* (Spirulinales, Figure 1M,N), and oscillatorialean taxa such as Gomontiellaceae (Bohunická et al., 2015a), *Desertifilum* (Dadheech et al., 2014; Sinetova et al., 2017), *Geitlerinema* and *Anagnostidinema* (Strunecký et al., 2017), *Jacksonvillea* (Hašler et al., 2017), *Wilmottia* (Strunecký et al., 2011), *Kamptonema* (Strunecký et al., 2014), and *Sodalinema* (Cellamare et al., 2018). Nevertheless, particular deviations of the parietal arrangement (Figure 5) may help characterizing individual taxa in combination with other traits.

Fascicular and irregular arrangements, initially described as a single “coiled (wavy)” type by Komárek and Kaštovský (2003), were split in our study based on the presence/absence of distinct thylakoid fascicles. The fascicular type seems to be rather universally adopted among the derived taxa with cell sizes >2.5 μm (Chroococcales, Pleurocapsales, Chroococcidiopsidales, part of Oscillatoriales and Nostocales), and therefore, like the parietal type, has limited taxonomic use. In Nostocales, the fascicular organization prevails in members of the rarely branching and akinete-forming family Nostocaceae (Flores and Herrero, 2010; Ramirez et al., 2011; Brito et al., 2012; Bohunická et al., 2015b; Gonzalez-Esquer et al., 2016), whereas the irregular type is found in Hapalosiphonaceae (Kaštovský and Johansen, 2008; Wilde et al., 2014; Gonzalez-Esquer et al., 2016), Symphyonemataceae (Lamprinou et al., 2013; Gonzalez-Esquer et al., 2016), Scytonemataceae (Fiore et al., 2007; Novis and Visnovsky, 2011; Lamprinou et al., 2012; Gonzalez-Esquer et al., 2016; Alvarenga et al., 2017), and Rivulariaceae (Brito et al., 2012; Alvarenga et al., 2016). However, neither these taxa are entirely monophyletic (Figure 8).

Although very conspicuous, radial thylakoid architecture does not serve as a suitable taxonomic marker. It seems to have serially emerged in a number of genera of the phylogenetically overlapping families Coleofasciculaceae (Casamatta et al., 2012, Figure 2A,B), Microcoleaceae (Porta et al., 2003; Chatchawan et al., 2012; Nguyen et al., 2013; Strunecký et al., 2013; Cellamare et al., 2018; Figure 2B,C), and Oscillatoriaceae (Ridley et al., 2005; Lokmer, 2007; Rasoulouniriana et al., 2009; Martins et al., 2016). In spite of a possible common tendency to form radial thylakoids in certain lineages, they usually occur in individual strains, intermixed with strains exhibiting other arrangements (Figure 8). Thus, these results do not support the original hypothesis of Komárek and Kaštovský (2003), which proposed the radial architecture as a synapomorphy of Phormidiaceae.

The parallel thylakoid architecture and the special type found in *Cyanothece sensu stricto* have each emerged only once, and therefore can serve as synapomorphies of Cyanobacteriaceae and Cyanothecaceae, respectively. This is in agreement with previous results on the phylogeny and ultrastructure of *Cyanobacterium*, *Cyanothece*, and *Geminocystis* (Komárek and Kaštovský, 2003; Komárek et al., 2004, 2014; Korelusová et al., 2009). Based on this, we can also suggest including *Geminocystis* in the family Cyanobacteriaceae.

The evolution of thylakoid organization roughly copies the phylogeny of housekeeping loci (Figure 8, 9). However, available evidence raises the possibility that certain architectures are to some extent also driven by cellular systems responsible for morphology, physiological/metabolic processes, and life strategy. This has been partly suggested by our morphometric analysis (Figure 7) and convergence of radial architecture in *Phormidium*-like cyanobacteria (Figure 8). Indeed, experimental studies documented profound effects of environmental factors, especially light conditions and nutrient starvation on thylakoid arrangement in both plants (Herbstová et al., 2012; Kirchhoff, 2013) and cyanobacteria (Barthel et al., 2013; Klotz et al., 2016).

This issue is closely related to the occurrence of special traits in cyanobacterial thylakoid architecture. For example, characteristic spherical formations have been recorded in cyanobacteria with both parietal (Taton et al., 2011; Bohunická et al., 2015a; Sinetova et al., 2017) and fascicular (Ramirez et al., 2011; Rigonato et al., 2016, this study) thylakoids. The spherical lamellae were documented to contain carboxysomes (Sinetova et al., 2017) and could possibly have physiological function linked to photosynthesis. In our study, an extreme case showing cells entirely filled with spherical formations was found in *Arthrospira* sp. PCC 8005 (Figure 3C,D), although typically, *Arthrospira* strains seem to have a thylakoid arrangement similar to the radial one (van Eykelenburg, 1979; Peduzzi et al., 2014; Cellamare et al., 2018). However, some lineages tend to contain spherical formations more than others (Figure 8). Similarly, a dense subperipheral layer of thylakoids was observed in *S. major* PCC 9313 (Figure 1M,N) while no such layer was found in *Spirulina* sp. by Palinska et al. (1998). Occasional formation of a single or few central thylakoid fascicles in cells with otherwise parietal thylakoid architecture sporadically occurs throughout the phylogenetic tree in medium-sized simple filamentous cyanobacteria (Strunecký et al., 2011, 2014; Dadheech et al., 2014; Mühlsteinová et al., 2014a,b; Cellamare et al., 2018). Perhaps, these strains with a common trichome width around 3 μm are at the very edge of morphologically limited parietal and fascicular organizations.

Finally, superficial analysis can distinguish special types that are consequence of the fact that standard TEM provides only a snapshot crossing a single cell in a single plane. An example of this probably is the triangular parietal arrangement documented in several taxa (Nierzwicki-Bauer et al., 1983; Guglielmi and Cohen-Bazire, 1984; Komárek and Kaštovský, 2003; Bruno et al., 2009; Strunecký et al., 2017; Zimba et al., 2017). In our opinion, it demonstrates a frequent presence of three peripheral fascicles of thylakoids in these clades. However, the same strains can also contain just two fascicles when viewed in a different TEM section (Nierzwicki-Bauer et al., 1983; Figure 1E,G).

### Auxiliary Analysis of Selected Proteins

We have not found any single protein whose phylogeny would be precisely in accordance with individual thylakoid types. This is however not extremely surprising. Functional variability of homologous proteins tends to be governed rather by their tertiary structure maintained by complementary changes, and by the replacement of individual ligand-binding residues rather than primary amino acid sequence used in standard phylogenetic analysis (Pils et al., 2005). Regulation of cellular processes at expression level and protein interactions may also be responsible for the observed variability in phenotypes. From this point of view, transcriptomic and proteomic analyses, and ultimately comparisons among structural models of the particular proteins are essential next steps in future research.

Another explanation, supported by our morphometric analysis (Figure 7), implies that cellular mechanisms, including various regulatory elements responsible for cell size and morphology affect also the morphology of thylakoids. Similarly, proteins regulating cell division of filamentous cyanobacteria could possibly influence the morphology of radial type of thylakoids. These possibilities are largely unexplored, and their investigation was beyond the scope of the current study.

Interestingly, homologs of several proteins postulated to be involved in thylakoid or chloroplast biogenesis and morphology were found only in limited number of cyanobacteria or were completely missing. For example, the thylakoid curvature protein CurT (Armbruster et al., 2013) was clearly missing in several basal cyanobacterial clades. As previously demonstrated, deletion in *curT* resulted in disrupted thylakoid organization and absence of biogenesis centers in *Synechocystis* (Heinz et al., 2016). Our results contradict the universal function of CurT in membrane architecture and suggest that CurT is not an universally essential factor for proper thylakoid biogenesis in cyanobacteria. Similarly, a TerC homolog can be found only in a very limited number of cyanobacteria, but remarkably including the thylakoid-less *Gloeobacter*. In *Arabidopsis*, TerC mutants are unable to accumulate newly synthesized thylakoid membrane proteins, which led to an assumption that TerC acts in insertion of thylakoid membrane proteins (Kwon and Cho, 2008; Theis and Schroda, 2016). In summary, it seems that the molecular mechanisms involved in thylakoid biogenesis and spatial organization substantially vary among cyanobacteria. Experimental discoveries made in plant chloroplasts or selected experimental strains of cyanobacteria can therefore rarely be generalized.

## Data Availability Statement

Raw datasets generated for statistical analysis and ancestral state reconstruction are included in the Supplementary Files. The phylogenetic trees based on selected proteins can be found in the Figshare digital repository using following permanent link: https://figshare.com/articles/supplementary_trees_Mares_etal_rar/7539875. Raw sequence alignment data supporting the conclusions of this manuscript will be made available by the authors, without undue reservation, to any qualified researcher.

## Author Contributions

JM and OS collected the data and performed the phylogenetic and bioinformatic analyses. JM, OS, and JW performed the electron microscopy analyses. JM performed the statistical analyses. LB and JM prepared the line drawings. JM, OS, and LB wrote the text.

## Conflict of Interest Statement

The authors declare that the research was conducted in the absence of any commercial or financial relationships that could be construed as a potential conflict of interest.

## Footnotes

1 blast.ncbi.nlm.nih.gov/Blast.cg
